# Mitochondrial Abnormality Facilitates Cyst Formation in Autosomal Dominant Polycystic Kidney Disease

**DOI:** 10.1128/MCB.00337-17

**Published:** 2017-11-28

**Authors:** Yu Ishimoto, Reiko Inagi, Daisuke Yoshihara, Masanori Kugita, Shizuko Nagao, Akira Shimizu, Norihiko Takeda, Masaki Wake, Kenjiro Honda, Jing Zhou, Masaomi Nangaku

**Affiliations:** a Division of Nephrology and Endocrinology, University of Tokyo Graduate School of Medicine, Tokyo, Japan; b Division of CKD Pathophysiology, University of Tokyo Graduate School of Medicine, Tokyo, Japan; c Education and Research Center of Animal Models for Human Diseases, Fujita Health University, Aichi, Japan; d Department of Analytic Human Pathology, Nippon Medical School, Tokyo, Japan; e Department of Cardiovascular Medicine, University of Tokyo Graduate School of Medicine, Tokyo, Japan; f Center for Polycystic Kidney Disease Research and Renal Division, Department of Medicine, Brigham and Women's Hospital, Harvard Medical School, Boston, Massachusetts, USA

**Keywords:** mitochondrial metabolism, polycystic kidney disease

## Abstract

Autosomal dominant polycystic kidney disease (ADPKD) constitutes the most inherited kidney disease. Mutations in the *PKD1* and *PKD2* genes, encoding the polycystin 1 and polycystin 2 Ca^2+^ ion channels, respectively, result in tubular epithelial cell-derived renal cysts. Recent clinical studies demonstrate oxidative stress to be present early in ADPKD. Mitochondria comprise the primary reactive oxygen species source and also their main effector target; however, the pathophysiological role of mitochondria in ADPKD remains uncharacterized. To clarify this function, we examined the mitochondria of cyst-lining cells in ADPKD model mice (Ksp-Cre *PKD1*^flox/flox^) and rats (Han:SPRD *Cy/+*), demonstrating obvious tubular cell morphological abnormalities. Notably, the mitochondrial DNA copy number and peroxisome proliferator-activated receptor γ coactivator 1α (PGC-1α) expression were decreased in ADPKD model animal kidneys, with PGC-1α expression inversely correlated with oxidative stress levels. Consistent with these findings, human ADPKD cyst-derived cells with heterozygous and homozygous *PKD1* mutation exhibited morphological and functional abnormalities, including increased mitochondrial superoxide. Furthermore, PGC-1α expression was suppressed by decreased intracellular Ca^2+^ levels via calcineurin, p38 mitogen-activated protein kinase (MAPK), and nitric oxide synthase deactivation. Moreover, the mitochondrion-specific antioxidant MitoQuinone (MitoQ) reduced intracellular superoxide and inhibited cyst epithelial cell proliferation through extracellular signal-related kinase/MAPK inactivation. Collectively, these results indicate that mitochondrial abnormalities facilitate cyst formation in ADPKD.

## INTRODUCTION

Autosomal dominant polycystic kidney disease (ADPKD) is a common genetic disorder that affects 12.5 million people worldwide in all ethnic groups and accounts for up to 10% of patients on renal-replacement therapy (1, 2). In this disease, progressive expansion of numerous bilateral renal cysts leads to massive kidney enlargement and progressive renal failure. ADPKD is caused by mutations in *PKD1* or *PKD2*, encoding the nonselective Ca^2+^ ion channels polycystin 1 and polycystin 2, respectively, which regulate Ca^2+^ influx in primary cilia (3). Lowered intracellular Ca^2+^-related abnormal signals lead to the induced proliferation of cyst epithelial cells arising from all nephron segments (4–6), which is a key feature associated with cyst formation. Reduced Ca^2+^ activates Ca^2+^-inhibitable adenylyl cyclase 6 (AC6) and increases the cyclic AMP (cAMP) level (7). In turn, enhanced cAMP and protein kinase A (PKA) signaling upregulates the extracellular signaling-regulated kinase (ERK) pathway in cells derived from polycystic kidneys (8). In this manner, ERK activation is known as a hallmark of ADPKD. Furthermore, the vasopressin 2 receptor (V2R) signaling pathway involves activation of a stimulatory G protein (Gs) followed by the activation of AC6 and the generation of the secondary messenger cAMP in cyst epithelial cells; accordingly, a V2R blocker is used for ADPKD treatment (9). Notably, *PKD1/2* deficiency impacts a variety of organs in addition to the kidneys, such that patients often present with extrarenal diseases involving the liver, heart, vasculature, and diverticulum (10).

The two-hit hypothesis is widely accepted in ADPKD cystogenesis, in which germ line and somatic mutations of two *PKD* alleles are necessary to initiate renal cyst formation (11, 12). However, the reason why numerous second hits occur in the kidneys of ADPKD patients is unclear. Recent clinical studies show that oxidative stress is present in early ADPKD, even when renal function is preserved (13, 14), suggesting that increased oxidative stress plays a functional role in cyst formation. As mitochondrial damage is a main trigger for intracellular superoxide generation, we hypothesized that *PKD1* mutations may occur in tubular cells harboring only a single wild-type allele in response to oxidative stress, leading to cyst formation. Therefore, in this study, we investigated the pathophysiological role of mitochondria in ADPKD.

## RESULTS

### Mitochondrial abnormalities in the kidneys of ADPKD animal models.

**FIG 1:**
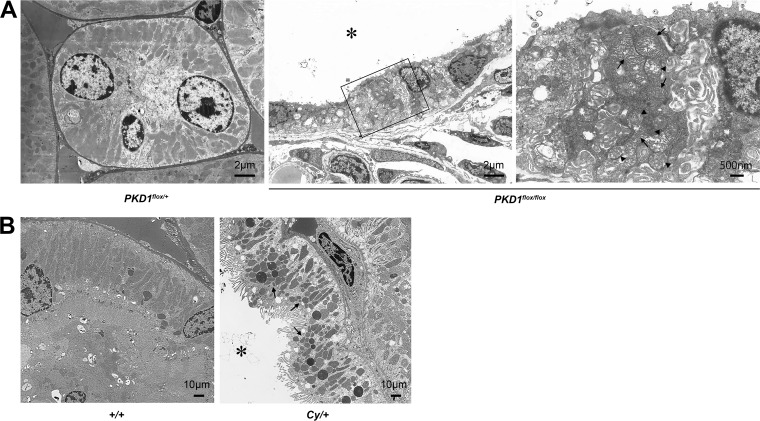
Morphological change of mitochondria in ADPKD animal models. (A) Electron microscope image of 7-day-old Ksp-Cre *PKD1*^flox/+^ mice and Ksp-Cre *PKD1*^flox/flox^ mice. Left, distal tubules of Ksp-Cre *PKD1*^flox/+^ mice. Middle, cyst epithelial cells from Ksp-Cre *PKD1*^flox/+^ mice; right, higher magnification of the middle panel. Arrows show normal mitochondria, and arrowheads show mitochondria that became swollen with indistinct cristae. *, cystic cavity. (B) Electron microscope images of proximal tubules from the kidneys of controls (*+/+*) and cysts derived from the proximal tubules of kidneys from *Cy/+* rats. The mitochondria of cyst-lining cells from *Cy/+* kidneys were fragmented (arrow). *, cystic cavity.

Ksp-Cre *PKD1*^flox/flox^ mice (15), as a rapid progression model of ADPKD, and heterozygous Han:SPRD Cy (*Cy/+*) rats (16), as a slow progression model of ADPKD, were used to assess disease-associated mitochondrial abnormalities in the kidney. Mitochondria are highly dynamic organelles that exhibit finely tuned and balanced frequencies of fusion and fission events to maintain homeostasis under physiological conditions. Alteration of mitochondrial morphology from an elongated network into small spheres or short rods is often accompanied by respiratory defects, increased production of mitochondrial reactive oxygen species ROS (mtROS), and oxidative damage (17), suggesting that proper mitochondrial morphology might be a protective factor involved in human disorders associated with mitochondrial DNA (mtDNA) mutations (18, 19). Therefore, we examined kidney tissues by transmission electron microscopy (TEM). Notably, the mitochondria of kidney cyst-lining cells from 7-day-old Ksp-Cre *PKD1*^flox/flox^ mice exhibited intermingled normal and abnormal shapes. Abnormally shaped mitochondria became swollen with indistinct and damaged cristae compared with those in normal distal tubular cells from control mice (Fig. 1A). Similarly, mitochondria of kidney cyst-lining cells from 7-week-old *Cy/+* rats were more fragmented than those of normal proximal-tubular epithelial cells from wild-type (*+/+*) rats (Fig. 1B).

**FIG 2:**
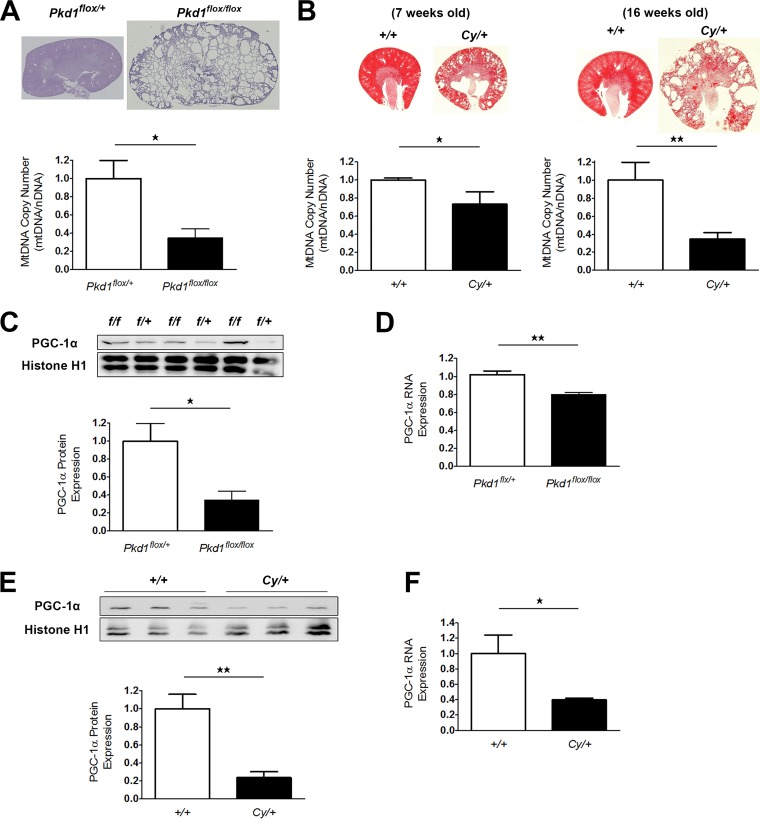
mtDNA copy number and PGC-1α expression in the kidneys of animal models of ADPKD. (A) Relative ratio of mtDNA copy number (mtDNA/nDNA) in kidney tissue from 7-day-old Ksp-Cre *PKD1*^flox/flox^ mice and controls (Ksp-Cre *PKD1*^flox/+^ mice). Top, representative kidney tissue of PAS staining. Bottom, Bar graph showing the relative ratio of mtDNA copy number (each group, *n* = 3). (B) Left, relative ratio of mtDNA copy number in kidney tissue from 7-week-old *Cy/+* rats and 7-week-old wild-type rats (*+/+*). Upper left, representative kidney tissue after hematoxylin-eosin (HE) staining. Lower left, bar graph showing the relative ratio of mtDNA copy number (each group, *n* = 3). Right, relative ratio of mtDNA copy number in kidney tissue from 16-week-old *Cy/+* rats and 16-week-old wild-type rats (*+/+*). Upper right, representative kidney tissue after HE staining. Lower right, bar graph showing the relative ratio of mtDNA copy number (each group, *n* = 3). (C) Representative Western blot analysis of PGC-1α in the kidneys of 7-day-old Ksp-Cre *PKD1*^flox/flox^ mice and controls (Ksp-Cre *PKD1*^flox/+^ mice). The bar graph shows the relative ratio of protein expression calibrated by histone H1 in control kidney tissue (each group, *n* = 3). (D) Representative real-time PCR analysis of mRNA for PGC-1α in the kidneys of 7-day-old Ksp-Cre *PKD1*^flox/flox^ mice and controls (each group, *n* = 3). (E) Representative Western blot analysis of PGC-1α in the kidneys of 7-week-old *Cy/+* rats and wild-type rats (*+/+*). (F) Representative real-time PCR analysis of mRNA for PGC-1α in the kidneys of 7-week-old *Cy/+* rats and wild-type rats (*+/+*) (each group, *n* = 3). The bar graph shows the relative ratio of protein expression calibrated by histone H1 in control kidney tissue. *, *P* < 0.05; **, *P* < 0.01.

Changes in mtDNA copy number are also a marker of mitochondrial abnormality (20). The mtDNA copy number in the kidney tissue of 7-day-old Ksp-Cre *PKD1*^flox/flox^ mice was markedly decreased compared to that in control animals (Fig. 2A). *Cy/+* rats also showed a copy number decrease at 7 weeks of age, which became more significant at 16 weeks (Fig. 2B). These results suggested that mitochondrial abnormality exists from an early phase of ADPKD and is related to disease progression.

**FIG 3:**
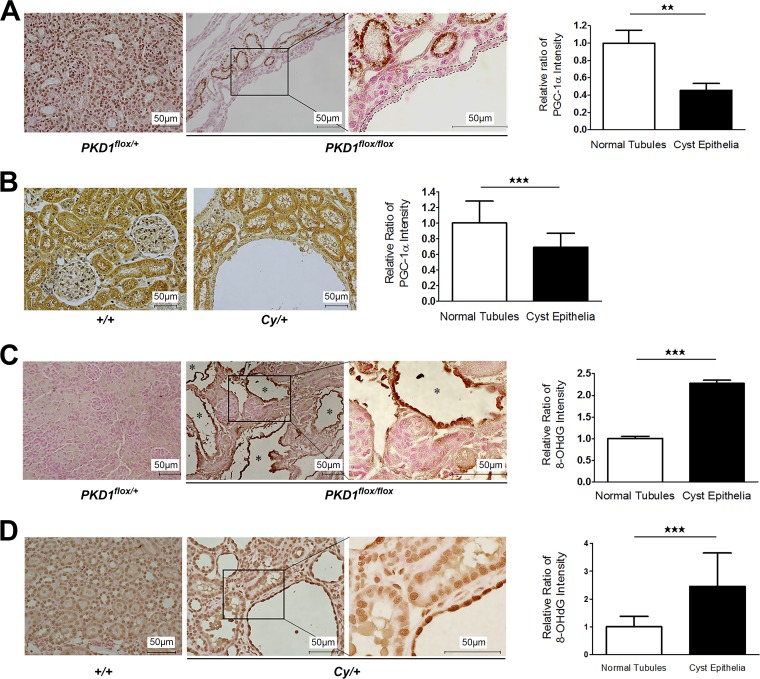
Immunohistochemical analysis of PGC-1α and 8-OHdG in the kidneys of ADPKD model animals. (A) Representative IHC staining for PGC-1α in kidney tissue from 7-day-old Ksp-Cre *PKD1*^flox/+^ and Ksp-Cre *PKD1*^flox/flox^ mice. Left, cortex of *PKD1*^flox/+^ mouse kidney; middle and right, lower and higher magnifications of *PKD1*^flox/flox^ mouse kidney, respectively. The area surrounded by the dotted line in the higher-magnification image represents cyst-lining cells. The bar graph shows the relative ratio of staining intensity of diaminobenzidine (DAB) in normal tubules and cyst-lining cells. More than 50 cells were analyzed for staining intensity of DAB in both groups. *, representative cyst. (B) Representative IHC staining for PGC-1α in kidney tissue from 7-week-old *Cy* rats. Left, IHC results for controls (*+/+*); right, IHC results for *Cy/+* rats. The bar graph shows the relative ratio of staining intensity of diaminobenzidine (DAB) in normal tubules and cyst-lining cells. More than 50 cells were analyzed for staining intensity of DAB in both groups. *, representative cyst. (C) Representative IHC staining results for 8-OHdG in kidney tissue from 7-day-old Ksp-Cre *PKD1*^flox/flox^ mice. Left, kidney tissue from Ksp-Cre *PKD1*^flox/+^ mice; middle and right, kidney tissue from Ksp-Cre *PKD1*^flox/flox^ mice and high magnification of the black box in the middle panel, respectively. The bar graph shows the relative ratio of staining intensity compared with that of normal tubules in Ksp-Cre *PKD1*^flox/flox^ mice. More than 50 cells were analyzed for staining intensity of DAB in both groups. *, representative cyst. (D) Representative IHC staining results for 8-OHdG in kidney tissue from 7-week-old *Cy* rats. *, representative cyst. The bar graph shows the relative ratio of staining intensity compared with that of normal tubules in *Cy* rats. The 8-OHdG staining intensity was increased in cyst-lining cells compared with normal tubular cells. More than 50 cells were analyzed for staining intensity of DAB in both groups. Results are shown as the relative ratio. Results represent the means ± standard deviations. **, *P* < 0.01; ***, *P* < 0.001.

Peroxisome proliferator-activated receptor γ coactivator 1α (PGC-1α) acts as a master regulator of mitochondrial biogenesis by regulating mitochondrial content (21); therefore, we assessed changes in PGC-1α expression in kidney tissue from ADPKD model animals to determine whether a lower renal mtDNA copy number was associated with decreased PGC-1α expression. As expected, PGC-1α protein and mRNA levels in the kidneys of Ksp-Cre *PKD1*^flox/flox^ mice (Fig. 2C and D) and 7-week-old *Cy/+* rats (Fig. 2E and F) were markedly lower than those in control animals. Subsequent immunohistochemical (IHC) analysis revealed a significant decrease in PGC-1α expression specifically in the kidney cyst-lining cells of ADPKD animals compared to the noncystic tubules of control animals (Fig. 3A and B), suggesting that mitochondrial abnormalities were present in the cyst-lining cells of ADPKD models. Reduced PGC-1α levels correlate with both decreased mtDNA copy number and the onset of oxidative stress (22, 23), and excessive mtROS production is a hallmark of mitochondrial dysfunction (24, 25). Moreover, IHC for the oxidative stress marker 8-hydroxy-2′-deoxyguanosine (8-OHdG) showed significant increases in marker expression in the kidney cyst-lining cells from Ksp-Cre *PKD1*^flox/flox^ mice and *Cy/+* rats (Fig. 3C and D), indicating that mitochondrial abnormalities likely incite oxidative stress in these cells. Together with the observed decrease in PGC-1α expression, these results suggest that mitochondrial abnormalities in the cyst-lining cells of ADPKD kidneys contribute to the induction of oxidative stress.

### Mitochondrial abnormalities in human cyst epithelial cells derived from an ADPKD patient with a *PKD1* homozygous mutation.

**FIG 4:**
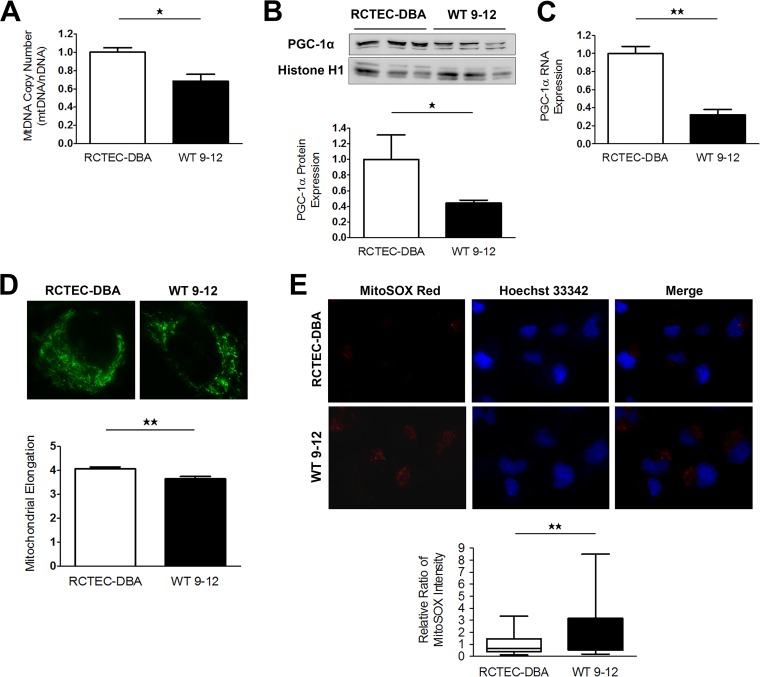
Mitochondrial abnormalities in human ADPKD cyst-derived cells with a homozygous *PKD1* mutation. (A) mtDNA copy number in cyst-derived cells containing a homozygous *PKD1* mutation (WT 9-12) compared to a normal tubular cell line (RCTEC-DBA) (each group, *n* = 3). Results represent the relative ratio. (B) Western blot analysis of PGC-1α levels in WT 9-12 and RCTEC-DBA (each group, *n* = 3). The bar graph shows the relative ratio of protein expression calibrated by histone H1 in normal and cyst-derived cells. (C) mRNA expression of PGC-1α in WT 9-12 and RCTEC-DBA (each group, *n* = 3). The bar graph shows the relative ratio of mRNA expression calibrated by GAPDH in normal and cyst-derived cells. (D) MitoTracker Red FM staining of WT 9-12 and RCTEC-DBA. Mitochondrial elongation was evaluated using ImageJ software, and units represent the ratio of major axis to minor axis (each group, *n* = 30). (E) Evaluation of mitochondrial superoxide in WT 9-12 compared to RCTEC-DBA by MitoSOX Red staining (each group, *n* = 25). The box plot shows the signal intensity. Results represent the means ± standard deviations. *, *P* < 0.05; **, *P* < 0.01.

**FIG 5:**
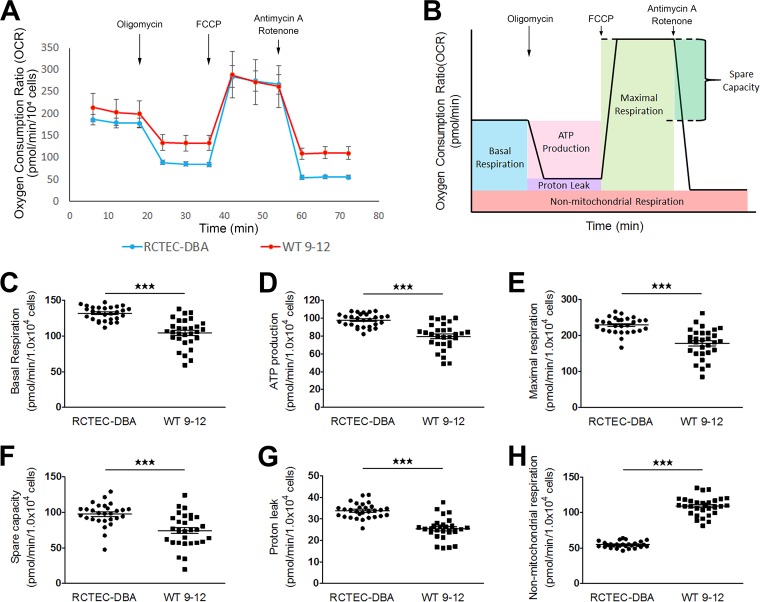
Alterations in mitochondrial metabolism in ADPKD-cyst-derived cells with a homozygous *PKD1* mutation. (A) Measurement of the mitochondrial OCR of normal tubular cells derived from distal tubules (RCTEC-DBA) and cyst-derived cells with a *PKD1* homozygous mutation derived from distal tubules (WT 9-12). (B) Seahorse XF Cell Mito stress test profile of the key parameters of mitochondrial respiration. (C to H) Basal respiration (C), ATP production with mitochondrial respiration (D), mitochondrial respiration and spare capacity (E and F, respectively), proton leakage from mitochondria (G), and nonmitochondrial respiration (H) in WT 9-12 compared to RCTEC-DBA. Each group, *n* = 30. Results represent the means ± standard deviations. ***, *P* < 0.001.

Our results from ADPKD model animals indicated that mitochondrial abnormalities may be present in cyst-lining cells. To investigate the pathogenic effect of mitochondrial abnormalities on cyst epithelial cells in human ADPKD, we performed *in vitro* studies using immortalized cyst-derived cells established from a single cyst obtained from distal cortical tubules collected from a patient with ADPKD harboring a homozygous *PKD1* mutation (WT 9-12) (26) and a normal human renal cortical tubular epithelial cell (RCTEC) line derived from normal distal tubule cells (RCTEC-Dolichos biflorus agglutin [RCTEC-DBA]). Similar to the *in vivo* data, the mtDNA copy number in cyst-derived cells harboring the homozygous *PKD1* mutation was significantly lower than that observed in normal cells (Fig. 4A). Additionally, PGC-1α protein and RNA expression in WT 9-12 was also reduced relative to that observed in RCTEC-DBA (Fig. 4B and C). Decreases in mDNA copy number and PGC-1α expression are associated with morphological changes, such as fragmentation, in mitochondria. Mitochondria from WT 9-12 were more fragmented than those from RCTEC-DBA, as estimated by MitoTracker Red FM staining (Fig. 4D). Furthermore, assays utilizing a mitochondrion-targeted fluorescent superoxide indicator revealed significantly increased superoxide levels in WT 9-12 compared with those observed in RCTEC-DBA (Fig. 4E), demonstrating that cyst epithelial cells produced excess superoxide owing to mitochondrial alterations. These results suggested that mitochondrial abnormality accompanied by increased mitochondrial superoxide exists in cyst-derived cells harboring the homozygous *PKD1* mutation. Furthermore, *PKD1^−/−^* mouse tubular cells are known to exhibit decreased oxidative phosphorylation (OXPHOS) activity as estimated by the oxygen consumption ratio (OCR) compared to normal controls (27–29), which was confirmed in the present study using an extracellular flux analyzer (Fig. 5A and B). Consistent with previous studies, except for nonmitochondrial respiration, the basal respiration, ATP production, maximal respiration, spare capacity, and proton leakage were decreased in human tubular cells with a homozygous *PKD1* mutation compared with those of the control (Fig. 5C to H). These results indicated that the number of mitochondria was also markedly reduced in the cells carrying the homozygous *PKD1* mutation.

### Mitochondrial abnormalities in human cyst epithelial cells derived from an ADPKD patient with a *PKD1* heterozygous mutation.

**FIG 6:**
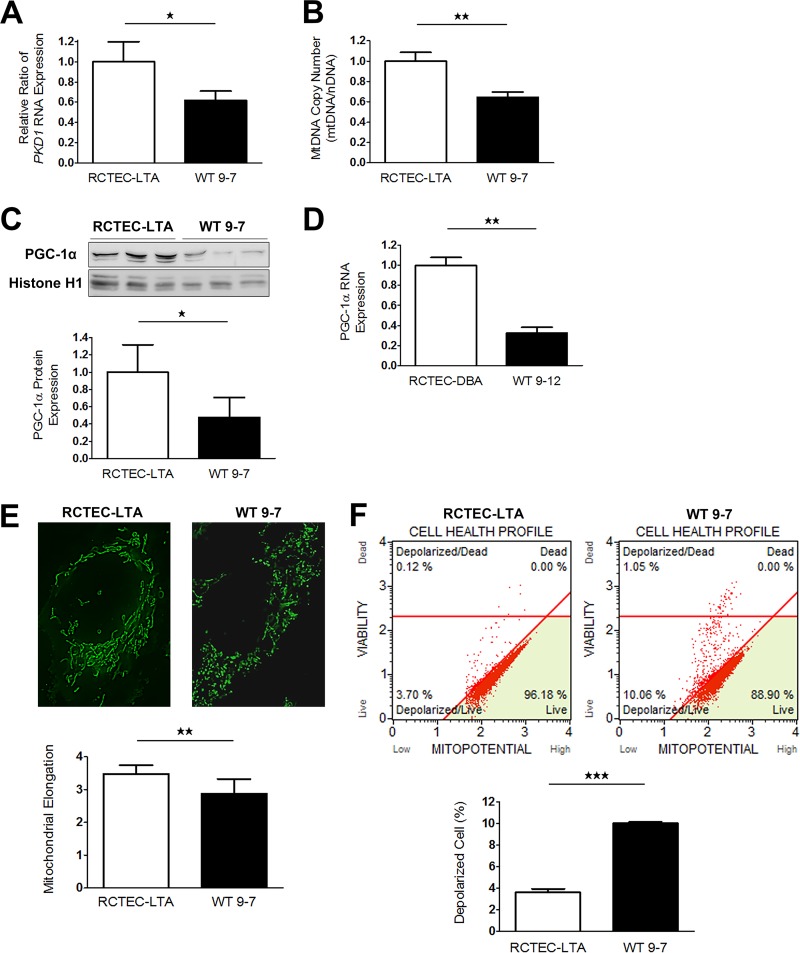

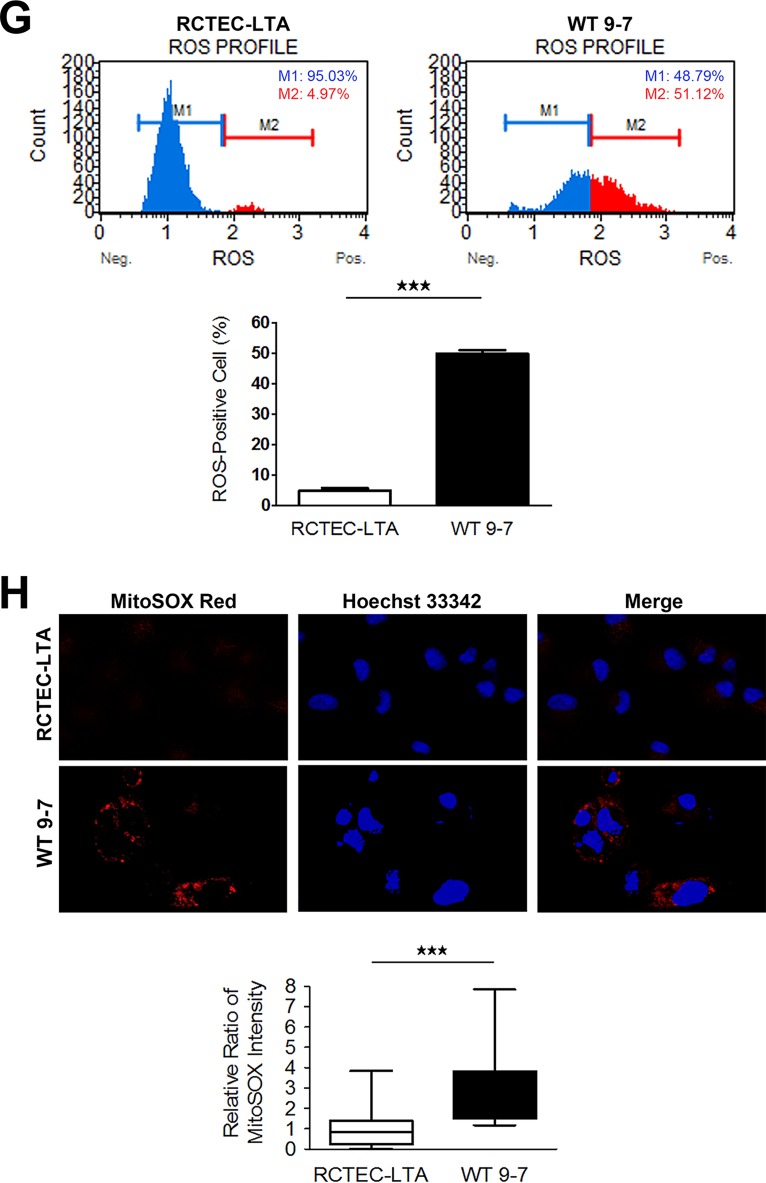
Mitochondrial abnormalities in human ADPKD cyst-derived cells with a heterozygous *PKD1* mutation. (A) *PKD1* expression in cyst-derived cells containing a heterozygous *PKD1* mutation (WT 9-7) compared to a normal tubular cell line (RCTEC-LTA) (each group, *n* = 3). Results represent the relative ratio. (B) mtDNA copy number in WT 9-7 relative to RCTEC-LTA (each group, *n* = 3). Results represent the relative ratio. (C) Western blot analysis of PGC-1α levels of WT 9-7 and RCTEC-LTA (each group, *n* = 3). The bar graph shows the relative ratio of protein expression calibrated by histone H1 in normal and cyst-derived cells. (D) mRNA expression of PGC-1α in WT 9-7 and RCTEC-LTA (each group, *n* = 3). The bar graph shows the relative ratio of mRNA expression calibrated by GAPDH in normal and cyst-derived cells. (E) MitoTracker Green staining of RCTEC-LTA and WT 9-7. Mitochondrial elongation was evaluated using ImageJ software, and units represent the major axis to minor axis ratio (each group, *n* = 25). (F) Evaluation of mitochondrial membrane potential. The bar graph shows the percentage of depolarized cells, showing depolarization of inner mitochondrial membrane potential in WT 9-7 compared with that in RCTEC-LTA (each group, *n* = 3). (G) Evaluation of intracellular ROS (superoxide). The *x* and *y* axes represent the cell number and the ROS signaling strength, respectively, with ROS-negative cells (M1) and ROS-positive cells (M2) identified. The ROS-positive cell number in WT 9-7 is compared with that in RCTEC-LTA. The bar graph shows the percentage of ROS-positive cells (each group, *n* = 3). (H) Evaluation of mitochondrial superoxide by MitoSOX Red staining. The box plot shows the signal intensity. Mitochondrial superoxide levels in WT 9-7 are compared to those in RCTEC-LTA (each group, *n* = 25). Results represent the means ± standard deviations. **, *P* < 0.01; ***, *P* < 0.001.

**FIG 7:**
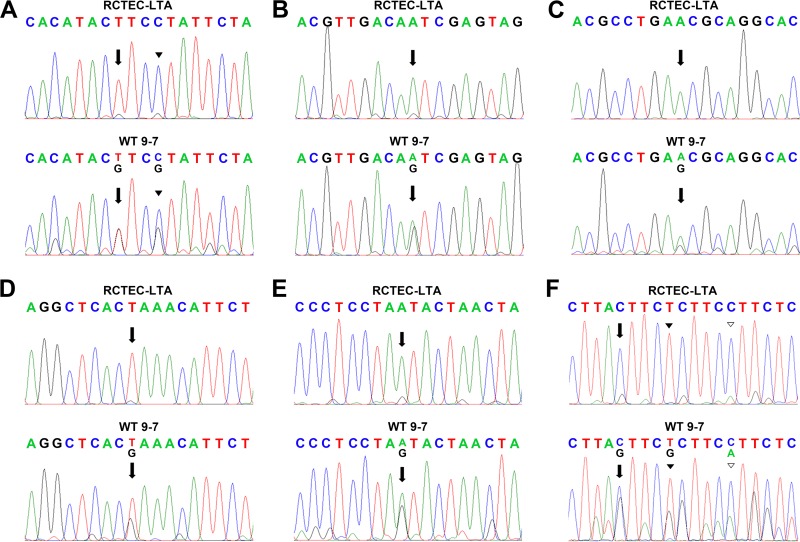
Comparison of mtDNA sequences in normal tubular cells (RCTEC-LTA) and cyst-derived cells with a heterozygous *PKD1* mutation (WT 9-7). Representative examples of mtDNA sequences, with accumulated mtDNA point mutations and heteroplasmy in cyst-derived cells, are shown. The influence of missense mutations on protein function was analyzed using polyPhen-2. (A) The region encoding NADH dehydrogenase subunit 4. The arrows point to mtDNA position 11204, and the arrowheads point to position 11207. A T-to-G replacement at position 11204 translates to a phenylalanine-to-valine mutation predicted to be benign (polyPhen-2 score, 0.035). A C-to-G replacement at position 11207 translates to a leucine-to-valine mutation predicted to be damaging (polyPhen-2 score, 0.994). (B) The region encoding cytochrome *c* oxidase subunit 2. The arrows show mtDNA position 8004. Replacement of A by G at position 8004 alters the amino acid asparagine to serine, and this mutation is predicted to be probably damaging, with a polyPhen-2 score of 0.973. (C) The region encoding NADH dehydrogenase subunit 4. The arrows show mtDNA position 11190. Replacement of A by G at position 11190 alters the amino acid asparagine to serine, which is predicted to be probably damaging, with a polyPhen-2 score of 0.994. (D) The region encoding NADH dehydrogenase subunit 4. The arrows show mtDNA position 11280. Replacement of T by G at position 11280 alters the amino acid leucine to arginine, which is predicted to be probably damaging, with a polyPhen-2 score of 0.999. (E) The region encoding NADH dehydrogenase subunit 4. The arrows show mtDNA position 11958. Replacement of A by G at position 11958 alters the amino acid methionine to serine, which is predicted to be benign, with a polyPhen-2 score of 0.121. (F) The region encoding cytochrome *b*. The arrows show mtDNA position 15443. Replacement of C by G at position 15443 alters the amino acid leucine to valine, which is predicted to be benign, with a polyPhen-2 score of 0.010. The black arrowhead shows mtDNA position 15447. Replacement of T by G at position 15447 alters the amino acid leucine to proline, which is predicted to be benign, with a polyPhen-2 score of 1.000. The white arrowhead shows mtDNA position 15452. Replacement of G by A at position 15452 alters the amino acid leucine to phenylalanine, which is predicted to be benign, with a polyPhen-2 score of 1.000.

To identify the time at which mitochondrial abnormality occurs in cyst-derived cells, we next used immortalized cyst-derived cells established from a single cyst obtained from proximal cortical tubules collected from a patient with ADPKD harboring a heterozygous *PKD1* mutation (WT 9-7) (26) and a normal human renal cortical tubular epithelial cell line derived from normal proximal tubule cells (RCTEC-Lotus tetragonolobus agglutinin [RCTEC-LTA]) to investigated this issue in further detail. *PKD1* mRNA expression decreased by approximately 50% in WT 9-7 compared to RCTEC-LTA (Fig. 6A). Consistent with the results for cyst-derived cells harboring a homozygous *PKD1* mutation, the mtDNA copy number (Fig. 6B) and PGC-1α protein and mRNA expression (Fig. 6C and D) in WT 9-7 were also lower than those observed in RCTEC-LTA, and mitochondria from WT 9-7 further showed more fragmented shapes than those from RCTEC-LTA (Fig. 6E). As depolarization of inner mitochondrial membrane potential is also a reliable indicator of mitochondrial dysfunction associated with increased mtROS production (30), we evaluated the mitochondrial membrane potential and discovered increased mitochondrial depolarization in WT 9-7 (Fig. 6F). This alteration in mitochondrial depolarization correlated with increased oxidative stress in cyst-derived cells, as estimated by intracellular ROS levels, which revealed a markedly higher percentage of ROS positivity in WT 9-7 than in RCTEC-LTA (Fig. 6G) in association with increased mitochondrial superoxide, the primary ROS in mitochondria (Fig. 6H). Because heteroplasmic mtDNA mutations constitute an index of increased ROS production (31), we analyzed the entire mtDNA sequence of WT 9-7 and found accumulated heteroplasmic mtDNA mutations in WT 9-7 (Fig. 7). Some of these heteroplasmic mtDNA mutations are predicted to severely influence each protein function and are also likely to cause mtROS production, agreeing with a previous study reporting that moderate amounts of mtDNA heteroplasmy promote tumorigenesis by increasing mtROS levels (32). These results are consistent with the previous findings suggesting that mitochondrial abnormality already existed in the cells with *PKD1* heteroplasmy and persists in the cells with *PKD1* homoplasmy.

### Enhanced PKA activity and mitochondrial oxygen consumption in the cyst-derived cells with *PKD1* heteroplasmic mutation.

**FIG 8:**
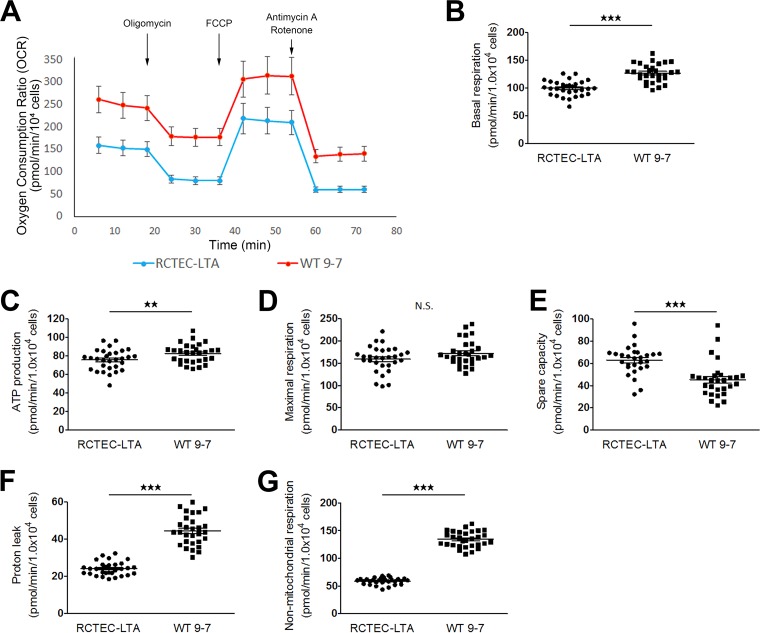
Alterations in mitochondrial metabolism in ADPKD-cyst-derived cells with a heterozygous *PKD1* mutation. (A) Measurement of the mitochondrial OCR of normal tubular cells derived from proximal tubules (RCTEC-LTA) and cyst-derived cells with a *PKD1* heterozygous mutation derived from proximal tubules (WT 9-7). (B to G) Basal mitochondrial respiration and ATP production (B and C, respectively), mitochondrial respiration (D), mitochondrial spare capacity (E), proton leakage from mitochondria (F), and nonmitochondrial respiration (G) in WT 9-7 compared with RCTEC-LTA. Each group, *n* = 30. Results represent the means ± standard deviations. **, *P* < 0.01; ***, *P* < 0.001. N.S., not significant.

**FIG 9:**
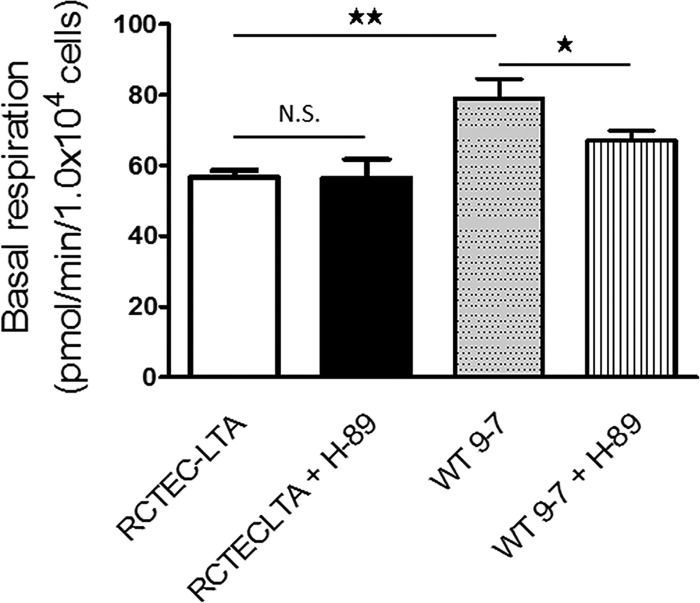
OCRs of normal tubular cells (RCTEC-LTA) and cyst-derived cells with a heterozygous *PKD1* mutation (WT9-7) treated with H-89 and analyzed by XF24. The OCR responses of normal tubular cells and cyst-derived cells in response to H-89 (PKA inhibition) are shown. Each group, *n* = 5. Results represent the means ± standard deviations. *, *P* < 0.05; **, *P* < 0.01. N.S., not significant.

The mitochondrial function of the cells with *PKD1* heteroplasmy was also investigated using the flux analyzer (Fig. 8A). Contrary to the results for cells with a homozygous *PKD1* mutation, basal respiration and ATP production were increased in WT 9-7 compared with RCTEC-LTA (Fig. 8B and C). The maximal mitochondrial respiration capacity did not differ between the groups (Fig. 8D), whereas the spare capacity of mitochondrial respiration was decreased in WT 9-7 (Fig. 8E). These results suggested that mitochondrial respiration was still maintained in cells with a heterozygous *PKD1* mutation. In comparison, a previous report showed that although mitochondrial dysfunction suppresses baseline OXPHOS activity in cells harboring mtDNA mutations, mitochondrial dysfunction also promotes excessive OXPHOS activity through cAMP-PKA activation (33). This observation may explain our results that increased basal respiration and ATP production but decreased spared capacity occurred in the cells with the heterozygous *PKD1* mutation with no difference in maximal mitochondrial respiration, because cAMP-PKA activity likely increased in the cells with the heterozygous *PKD1* mutation. Increased proton leakage (Fig. 8F) is further suggestive of mitochondrial abnormality in the cells with the heterozygous *PKD1* mutation. Notably, increased nonmitochondrial respiration was also found in the cells with the heterozygous *PKD1* mutation (Fig. 8G), as was found in the cells with the homozygous *PKD1* mutation; these data suggest that not only mitochondrial superoxide but also nonmitochondrial ROS will increase in cyst-derived cells compared with normal tubular cells. To confirm the effect of cAMP-PKA in mitochondrial respiration, RCTEC-LTA and W 9-7 were treated with a PKA inhibitor (H-89), which demonstrated that H-89 reduced basal respiration of WT 9-7 but not RCTEC-LTA (Fig. 9). Together with the findings from the investigations in cells with a heterozygous *PKD1* mutation, this result suggests that the mitochondria of cyst-derived cells with a *PKD1* heteroplasmic mutation are not normal but exhibit cAMP-PKA-stimulated mitochondrial oxygen consumption. This in turn will enhance superoxide production in the mitochondria of these cells.

### *PKD1* affects mitochondrial functions in renal tubular cells in a dose-dependent manner.

**FIG 10:**
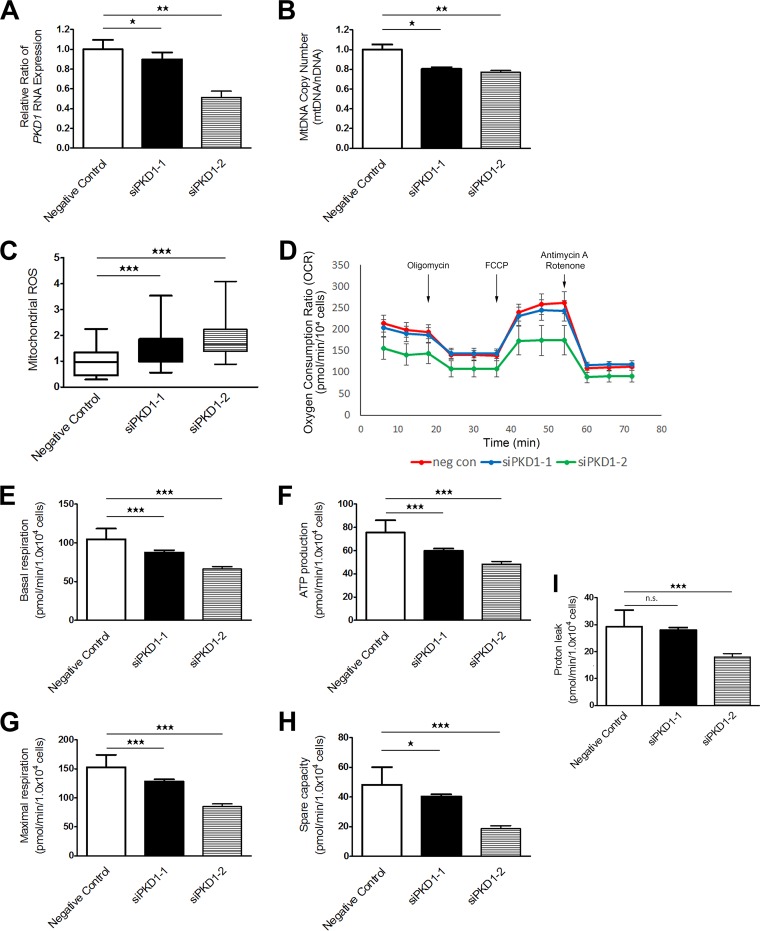
RNA interference-mediated *PKD1* knockdown and mitochondrial abnormality. Two kinds of si*PKD1* (si*PKD1*-1 and si*PKD1*-2) were used. (A) *PKD1* knockdown efficiency confirmed by quantitative real-time PCR. The bar graph shows the relative ratio of *PKD1* RNA expression (each group, *n* = 3). (B) Evaluation of mtDNA copy number by real-time PCR in association with *PKD1* RNA expression levels (each group, *n* = 3). Results represent the relative ratio. (C) Evaluation of mitochondrial superoxide by MitoSOX Red staining as correlated with *PKD1* RNA expression levels (each group, *n* = 25). The box plot shows the signal intensity. (D) Alterations in mitochondrial metabolism in PKD1 knockdown cells. Red, blue, and green represent negative-control siRNA, si*PKD1*-1, and si*PKD1*-2, respectively (each group, *n* = 20). (E to I) Basal respiration (E), ATP production with mitochondrial respiration (F), mitochondrial respiration (G), spare capacity (H), and proton leakage from mitochondria (I) as correlated with *PKD1* RNA expression levels. Results represent the means ± standard deviations. *, *P* < 0.05; **, *P* < 0.01; ***, *P* < 0.001; n.s., not significant.

To confirm the relationship between *PKD1* and mitochondrial function, we analyzed mitochondrial functions in cells with *PKD1* knockdown. Here we knocked down *PKD1* mRNA in RCTEC-LTA by using *PKD1*-specific small interfering RNAs (siRNAs) (si*PKD1*-1 and si*PKD1*-2). The efficiency of knockdown of *PKD1* was confirmed with quantitative real-time PCR (Fig. 10A). As expected, the mtDNA copy number was decreased (Fig. 10B) and mitochondrial superoxide production was increased (Fig. 10C) in the cells with *PKD1* knockdown. In addition, the degree of *PKD1* mRNA expression correlated with mtDNA copy number and inversely correlated with mitochondrial ROS production. Mitochondrial function was also analyzed in the cells with *PKD1* knockdown using the extracellular flux analyzer (Fig. 10D), which demonstrated that all parameters associated with mitochondria decreased according to *PKD1* expression level (Fig. 10E to I). These data indicate that *PKD1* expression is very important for normal mitochondrial function.

### Signaling pathways contributing to mitochondrial abnormalities.

**FIG 11:**
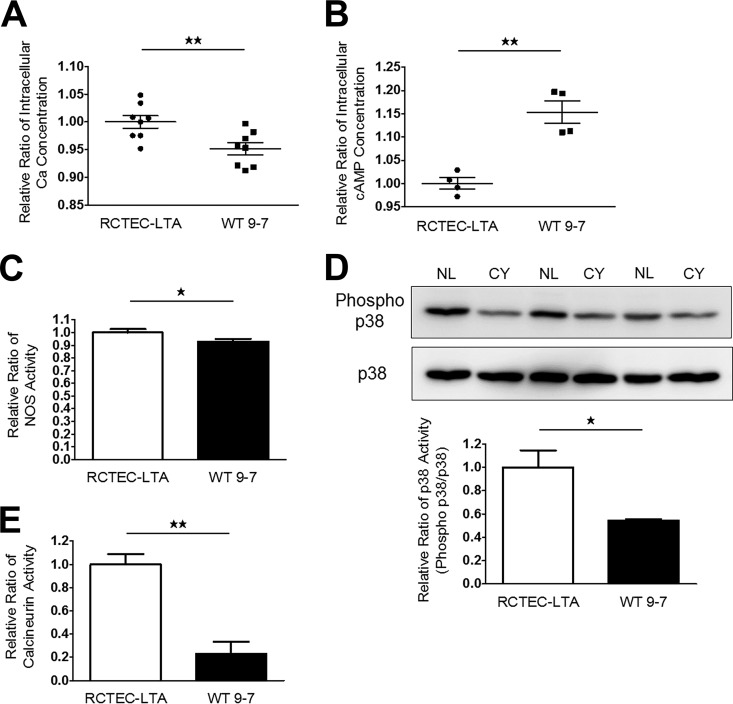
PGC-1α regulatory mechanisms in cyst-derived cells. (A and B) Intracellular Ca^2+^ (each group, *n* = 8) (A) and cAMP concentrations (each group, *n* = 4) (B) of normal tubular cells (RCTEC-LTA) and cyst-derived cells (WT 9-7). Results represent the relative ratio. (C and D) NOS (C) and p38 MAPK (D) activities of RCTEC-LTA and WT 9-7 (each group, *n* = 3). Results represent the relative ratio. The bar graph shows the relative ratio of p38 activity (phosphor-p38/p38). (E) Calcineurin activities of RCTEC-LTA and WT 9-7 (each group, *n* = 3). Results represent the relative ratio. All results represent the means ± standard deviations. *, *P* < 0.05; **, *P* < 0.01.

Although defective *PKD1* expression and PGC-1α expression are expected to induce subsequent functional and morphological alterations in mitochondria, as well as increased mitochondrial superoxide, in cyst epithelial cells, the signaling mechanisms associated with reduced PGC-1α expression remain unclear. Therefore, we assessed the regulation of PGC-1α expression and mitochondrial biogenesis by signaling pathways. The Ca^2+^ signaling pathway is known to induce PGC-1α expression (34) via a variety of signaling mechanisms, including adrenergic/cAMP (35), nitric oxide-soluble guanylate cyclase (36), calcineurin (34), and p38 mitogen-activated protein kinase (MAPK), which have previously been shown to upregulate PGC-1α expression (37). However, decreased intracellular Ca^2+^ has been found in MDCK cells with heterologous expression of polycystin 1 (38), and intracellular Ca^2+^ concentrations in WT 9-7 have not been reported. Therefore, we confirmed the presence of decreased intracellular Ca^2+^ concentrations (Fig. 11A) and also increased cAMP levels (Fig. 11B), both of which are pathogenic properties of ADPKD in cyst-derived cells. We also observed that regulators of PGC-1α expression, including nitric oxide synthase (NOS) activity (Fig. 11C) and the activities of the Ca^2+^-related molecules p38 MAPK (Fig. 11D) and calcineurin (Fig. 11E), were lower in cyst-derived cells. This relationship between a Ca^2+^-related signaling pathway and PGC-1α is plausible, given that ADPKD is a disease associated with Ca^2+^ channel dysfunction.

### The mitochondrion-specific antioxidant MitoQ reduces mitochondrial superoxide production and proliferation of cyst-derived cells.

**FIG 12:**
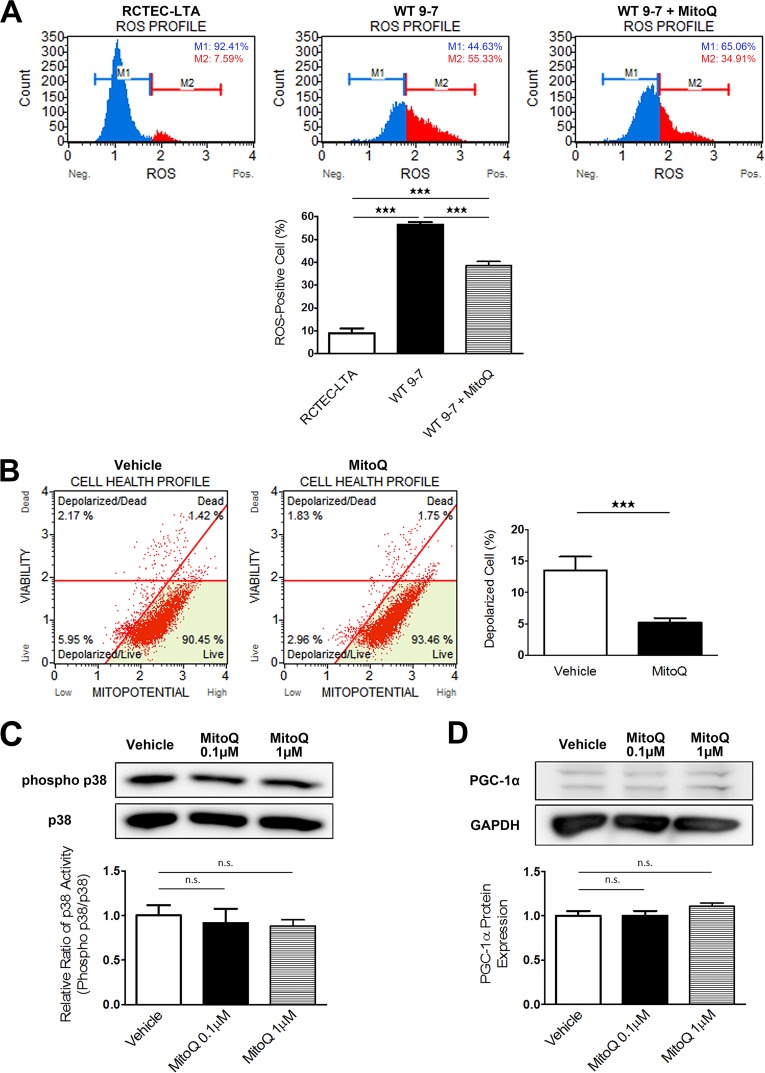

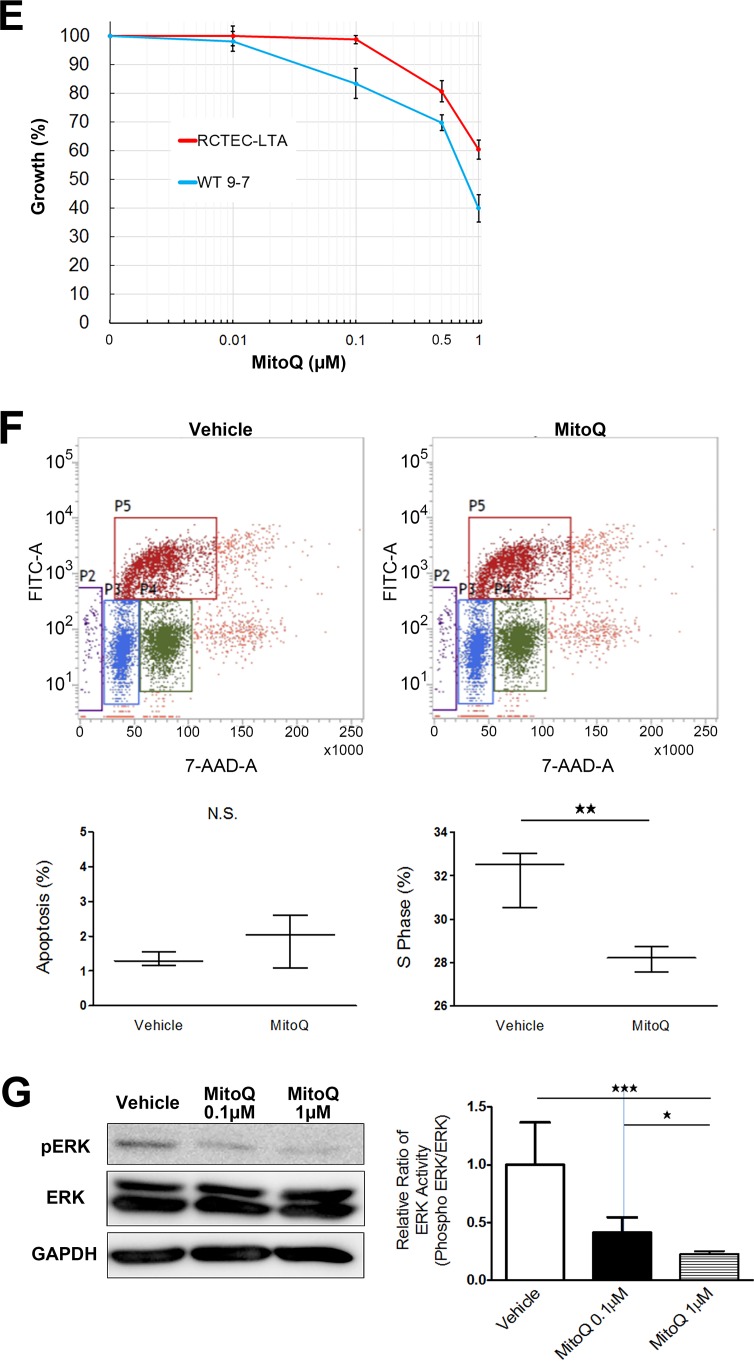
Amelioration of mitochondrial abnormality in cyst-derived cells by mitochondrion-targeted therapy (MitoQ). (A) Evaluation of intracellular ROS (superoxide) to assess intracellular superoxide levels following mitochondrion-targeted therapy using MitoQ. The *x* and *y* axes represent cell number and ROS signaling strength, respectively, with ROS-negative cells (M1) and ROS-positive cells (M2) identified. The ROS-positive cell number in cyst-derived cells (WT 9-7) are compared with that in control cells (RCTEC-LTA) and MitoQ-treated (1.0 μM for 48 h) cyst-derived cells (Cyst + MitoQ). The bar graph shows the percentage of ROS-positive cells (each group, *n* = 3). (B) Evaluation of mitochondrial membrane potential. The bar graph shows the percentage of depolarized cells (each group, *n* = 3). Depolarization of inner mitochondrial membrane potential in cyst-derived cells treated with MitoQ (1.0 μM for 48 h) (MitoQ) compared with cyst-derived cells treated with vehicle (Vehicle) is shown. (C) p38 MAPK activities in WT 9-7 treated with MitoQ (each group, *n* = 3). Results represent the relative ratio. The bar graph shows the relative ratio of p38 activity (phosphor-p38/p38). (D) PGC-1α expression in WT 9-7 treated with MitoQ (each group, *n* = 3). Results represent the relative ratio. The bar graph shows the relative ratio of PGC-1α expression. (E) Effect of MitoQ treatment (48 h) on cell proliferation of RCTEC-LTA and WT 9-7 (each group, *n* = 3). (F) Results of BrdU flow cytometry regarding the percentages of apoptotic and S-phase cells in control and MitoQ-treated groups (each group, *n* = 3). (G) Western blot analysis of ERK showing effects of MitoQ treatment on ERK activity (each group, *n* = 3). Results represent the means ± standard deviations. *, *P* < 0.05; **, *P* < 0.01; ***, *P* < 0.001. n.s., not significant.

MitoQuinone (MitoQ) is a well-characterized, mitochondrion-targeted antioxidant that accumulates near the mitochondrial inner membrane to protect it from lipid peroxidation (39). To evaluate the pathophysiological role of mitochondrial abnormalities in cyst-derived cells with a heterozygous *PKD1* mutation, we treated WT 9-7 with MitoQ and evaluated superoxide levels. Treatment of WT 9-7 with MitoQ for 24 h reduced intracellular superoxide levels (Fig. 12A) and ameliorated mitochondrial depolarization (Fig. 12B). In particular, we showed that these effects were not due to p38 phosphorylation or PGC-1α expression-mediated mechanisms: MitoQ did not affect the p38 activity or PGC-1α expression level (Fig. 12C and D). Abnormal proliferation of cyst-lining cells is important for cyst formation, and cyst-derived cells from ADPKD kidneys exhibit increased activity in the ERK/MAPK pathway (40), which constitutes a key signaling cascade involved in regulating multiple processes required for cell proliferation. Furthermore, mtROS are important for ERK/MAPK signaling in tumor cells (41). We observed that MitoQ treatment reduced cyst-derived cell proliferation in a dose-dependent manner and that this effect was more significant in WT 9-7 than in RCTEC-LTA (Fig. 12E). This inhibitory effect occurs through the mechanism of cell cycle arrest (Fig. 12F) consequent to the inactivation of ERK-regulated cell proliferation signals (Fig. 12G) rather than by an increase in apoptosis (Fig. 12F). These results demonstrated that mitochondrion-derived oxidative stress likely represents an important factor for cyst epithelial cell proliferation in ADPKD.

## DISCUSSION

In this study, mitochondrial morphological changes in the kidneys of ADPKD model animals led us to determine possible abnormalities, including disruptions in Ca^2+^ influx and increased cAMP concentrations induced by polycystin dysfunction, which play a central role in ADPKD pathogenesis (42). As previously reported, elevated cytosolic Ca^2+^-mediated PGC-1α activation is important to increase mitochondrial biogenesis (43). Additionally, increased cAMP levels activate PKA, which enhances mitochondrial respiration (44), whereas PKA-mediated MIC60 phosphorylation negatively regulates the clearance of abnormal mitochondria (45). Therefore, an accumulation of mitochondrial abnormalities would be expected in ADPKD pathogenesis. Here, we identified that mitochondrial abnormalities exist not only in the cells with a homozygous *PKD1* mutation but also in those carrying a heterozygous *PKD1* mutation. Consistent with previous studies showing that *PKD1* and *PKD2* haploinsufficiency reduces intracellular Ca^2+^ levels (46, 47), this reduction of intracellular Ca^2+^ might result in the introduction of mitochondrial abnormalities according to the mechanisms outlined here. Notably, owing to the lack of introns and protective histones, mtDNA is 10- to 20-fold more vulnerable to oxidative damage and subsequently more prone to mutation than nuclear DNA (nDNA) (48). Additionally, the frequency of mtDNA mutations is tissue specific and markedly increased in the kidney and liver compared with that in other human organs (49). Our data indicate that lower Ca^2+^-related mitochondrial abnormality will initiate from the stage of heterozygous mutation of the *PKD1* gene. In fact, tubular cells with heterozygous mutation of *PKD1* exhibited accumulated mtDNA mutations and increased mitochondrial superoxide compared with normal tubular cells.

Furthermore, a recent study demonstrated the importance of PGC-1α in the pathogenesis of kidney disease (22); in addition, PGC-1α is required for the induction of many ROS-detoxifying enzymes (50). Together with our data, these data indicate that reduced PGC-1α is associated with not only enhancement of mitochondrial superoxide but also reduction of the antioxidative effect, which combined might underlie the increased oxidative stress observed in ADPKD. Oxidative DNA damage is a major cause of DNA mutations, with oxidative activity also being capable of inducing spontaneous mutagenesis in mammalian cells (51). We propose the possibility that mitochondrial abnormalities accumulate in tubular cells of patients with ADPKD carrying heterozygous *PKD1* mutations because of decreased intracellular Ca^2+^ levels and that excessive superoxide from abnormal mitochondria in the kidneys of patients with ADPKD causes DNA damage, which includes second mutation of *PKD*-related genes. This might also explain the high risk of development of a variety of cancers in patients with ADPKD (52), given the necessity of DNA damage for carcinogenesis to occur. Notably, this hypothesis might explain why oxidative stress is evident in patients with early-stage ADPKD, even in those with preserved kidney function (13, 14). Furthermore, ROS also play critical roles in regulating cell metabolism, with increased mtROS levels inducing cell proliferation (53). As cell proliferation is an important factor in ADPKD cystogenesis, inhibition of mitochondrial superoxide might constitute an effective strategy for the treatment of patients with early-stage ADPKD.

A limitation of this study is that we were unable to investigate human ADPKD kidney tissues; therefore, it could not be confirmed that mitochondrial abnormality exists in the tubular cells prior to cyst formation (before the second hit of *PKD* gene mutation). Further studies are therefore needed to demonstrate this phenomenon directly in patients.

**FIG 13:**
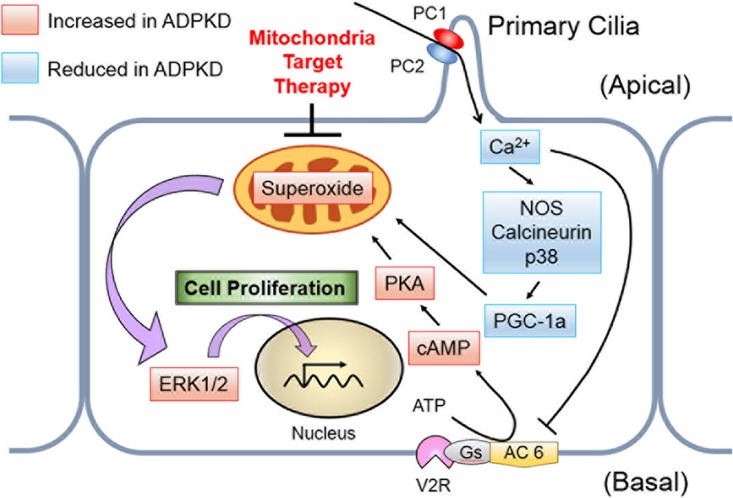
Schematic of the pathway proposed in this study. A decreased intracellular Ca^2+^ concentration reduces PGC-1α expression via calcineurin, p38 MAPK, and NOS deactivation, whereas PKA upregulates mitochondrial respiration. These mechanisms enhance mitochondrial superoxide production, which contributes to ERK/MAPK signaling in cyst epithelial cells. PC 1, polycystin 1; PC 2, polycystin 2; AC 6, adenylate cyclase 6; V2R, vasopressin-2 receptor; Gs, stimulatory G protein.

The field of mitochondrial biology has progressed substantially in recent years and has yielded numerous opportunities to translate discoveries, including MitoQ, to clinical medicine (54). Our findings offer valuable insight into new avenues for novel therapeutic approaches toward ADPKD treatment, including mitochondrion-targeted therapies as viable options (Fig. 13).

## MATERIALS AND METHODS

### ADPKD animal models.

The Ksp-Cre *PKD1*^flox/flo*x*^ mouse was established by Shibazaki et al. as an ADPKD model animal (15). In this model, *PKD1* expression was selectively knocked out in the thick ascending limb through the collecting duct in the kidney. Ksp-Cre *PKD1*^flox*/+*^ mice do not exhibit any renal cysts and are used as normal controls of Ksp-Cre *PKD1*^flox/flox^ mice. Kidney samples from 7-day-old Ksp-Cre *PKD1*^flox/flox^ mice (*n* = 3), with samples from Ksp-Cre *PKD1*^flox/+^ mice (*n* = 3), were kindly provided by S. Nishio of the Hokkaido University Graduate School of Medicine (Hokkaido, Japan). The Han:SPRD-*Cy* (*Cy*) rat strain was discovered by Kaspareit-Rittinghausen et al. (55) as a spontaneous hereditary model that closely resembles human ADPKD. This model is characterized by a slow progression. Kidney samples of 7- and 16-week-old Han:SPRD-*Cy* rats were provided by S. Nagao of Fujita Health University (Aichi, Japan). Here, we used wild-type (*+/+*) (*n* = 3) and heterozygous (*Cy/+*) (*n* = 3) rats. All protocols for animal experiments were approved by the Ethical Committee on Animal Experiments of the University of Tokyo (M-P16-082), and all animal experiments were conducted in accordance with institutional guidelines.

### Cell culture.

Loghman-Adham et al. (56) developed immortalized cells from the cyst epithelia of individual cysts from human patients with ADPKD and a control cell line from normal human renal cortex. *PKD1* mutation-containing immortalized cyst-derived cells WT9-7 (CRL-2830; American Type Culture Collection, Manassas, VA) and WT 9-12 (CRL-2833; American Type Culture Collection) were used as cyst epithelial cells. WT 9-7 is of cyst proximal tubule origin, and WT 9-12 is of cyst distal tubule origin. The heterozygous *PKD1* mutation in WT 9-7 and homozygous *PKD1* mutation in WT 9-12 were reported by Nauli et al. (26). Immortalized normal human RCTECs derived from proximal tubules (RCTEC-Lotus tetragonolobus agglutinin [RCTEC-LTA]) and distal tubules (RCTEC-Dolichos biflorus agglutin [RCTEC-DBA]) (56) were used for *in vitro* studies as normal controls. Both cell lines were grown at 37°C in 95% air–5% CO_2_ in Dulbecco's modified Eagle's medium (DMEM) (D6429; Sigma-Aldrich, St. Louis, MO) supplemented with 10% fetal bovine serum (Sigma-Aldrich) in flasks.

### DNA isolation and mtDNA copy number analysis.

Assessment of human and rat mtDNA copy numbers by quantitative real-time PCR was performed as previously described (57). Total DNA was isolated from whole kidneys and normal and cystic tubular cells using a NucleoSpin tissue kit (TaKaRa Bio, Otsu, Japan). DNA (10 ng) was subjected to quantitative real-time PCR on a CFX96 system (Bio-Rad, Hercules, CA) with Kapa SYBR fast universal 2× quantitative PCR (qPCR) master mix (Kapa Biosystems, Wilmington, MA). The PCR protocol consisted of 95°C for 30 s followed by 40 cycles of 95°C for 5 s, annealing at 60°C for 30 s, and a 72°C extension for 10 s. Human and rat mtDNA copy numbers and nDNA content were determined by amplifying a short region of the *MT-TL1* (tRNA-Leu^UUR^) and *B2M* genes (58), respectively, whereas those in rats were assessed by amplifying *Mt-cyb* and *Actb*, respectively (59). The mtDNA copy number was then calculated according to the mtDNA/nDNA ratio. All primers are listed in Table 1.

**TABLE 1:** Primers for analysis of mtDNA copy number

Species	Genome	Target	Primer sequence, 5′→3′	
			Forward	Reverse
Rattus norvegicus	Mitochondrial	Cytochrome *b*	GCAGCTTAACATTCCGCCCAATCA	TACTGGTTGGCCTCCGATTCATGT
	Nuclear	Actin	ATCATGTTTGAGACCTTCAACACCC	CATCTCTTGCTCGAAGTCTAGG
Mus musculus	Mitochondrial	tRNA-Val	CTAGAAACCCCGAAACCAAA	CCAGCTATCACCAAGCTCGT
	Nuclear	β2-Microglobulin	ATGGGAAGCCGAACATACTG	CAGTCTCAGTGGGGGTGAAT
Homo sapiens	Mitochondrial	tRNA-Leu	CACCCAAGAACAGGGTTTGT	TGGCCATGGGTATGTTGTTA
	Nuclear	β2-Microglobulin	TGCTGTCTCCATGTTTGATGTATCT	TCTCTGCTCCCCACCTCCAAGT

### mRNA extraction and expression analysis.

mRNAs were isolated with RNAiso Plus (TaKaRa Bio). Reverse transcription was performed using PrimeScript RT master mix (TaKaRa Bio). Primer pairs designed to generate overlapping PGC-1α (PPARGC1A), *PKD1*, and glyceraldehyde-3-phosphate dehydrogenase (GAPDH) fragments are listed in Table 2. We used 10 ng of mtDNA for quantitative real-time PCR using Kapa SYBR fast universal 2× qPCR master mix (Kapa Biosystems) run on a CFX96 system (Bio-Rad). The PCR protocol consisted of 95°C for 30 s followed by 40 cycles at 95°C for 5 s, annealing at 62°C for 30 s, and a 72°C extension for 5 s, accompanied by real-time data collection. The relative ratios of mRNA of PGC-1α and *PKD1* expression were calibrated by GAPDH.

**TABLE 2:** Primers for analysis of PGC-1α RNA expression

Species	Target	Primer sequence, 5′→3′	
		Forward	Reverse
Rattus norvegicus	GAPDH	CAACTCCCTCAAGATTGTCAGCAAGGCAT	GGCATGGACTGTGGTCATGA
	PGC-1α	GAAAAAGCTTGACTGGCGTC	GCAGCACACTCTATGTCACTC
Mus musculus	GAPDH	CATGGCCTTCCGTGTTCCTA	CCTGCTTCACCACCTTCTTGAT
	PGC-1α	GAAAAAGCTTGACTGGCGTC	GCAGCACACTCTATGTCACTC
Homo sapiens	GAPDH	CCTCAACGACCACTTTGTCA	TTACTCCTTGGAGGCCATGT
	PGC-1α	GACACCCTCTTCTCTTCCTTCTTT	CGGCTGTTACTCTCTCTCCTTG

### Antibodies.

Mouse monoclonal anti-GAPDH antibody (sc-32233), rabbit polyclonal anti-ERK1 antibody sc-94), mouse monoclonal anti-pERK antibody (sc-7383), and mouse monoclonal anti-histone H1 antibody (sc-8030) were purchased from Santa Cruz Biotechnology (Dallas, TX) and used for Western blot (WB) analysis. Anti-PGC-1α antibody (ab54481) was obtained from Abcam (Cambridge, UK) and used for WB and IHC analyses. Anti-8-OHdG antibody (MOG-020P) was obtained from the Japan Institute for the Control of Aging (Shizuoka, Japan). Rabbit polyclonal anti-p38 MAPK antibody and rabbit polyclonal anti-phospho-p38 MAPK were obtained from Cell Signaling Technology, Japan (Tokyo, Japan). Horseradish peroxidase (HRP)-conjugated goat anti-mouse IgG (170-6515; Bio-Rad) and HRP-conjugated donkey anti-goat IgG (sc-2020; Santa Cruz Biotechnology) were used as secondary antibodies for WB analysis.

### WB analysis.

Nuclear extracts from cultured cells or animal kidney tissue were obtained using an NE-PER nuclear and cytoplasmic extraction kit (Thermo Fisher Scientific, Waltham, MA), and the protein concentration was measured using a Pierce bicinchoninic acid (BCA) protein assay kit (Thermo Fisher Scientific). For other Western blot (WB) analyses, whole-cell lysates of cultured cells or animal kidney tissue were obtained using a nuclear extract kit (Active Motif, Carlsbad, CA). Sodium dodecyl sulfate (SDS) sample buffer containing 0.70 M Tris-HCl (pH 6.8), 20% SDS, 72% glycerol, 10% β-mercaptoethanol, and 0.024% bromophenol blue was added to the lysate and then separated on 10% SDS-polyacrylamide gels. After electrophoresis, proteins were transferred to polyvinylidene difluoride membranes (GE Healthcare, Little Chalfont, UK) in a Tris-glycine transfer buffer (48 mM Tris buffer, 39 mM glycine, 0.05% SDS, and 10% methanol). Membranes were incubated with primary and secondary antibodies, and an ECL Plus Western blot system (GE Healthcare) was used to detect immunoreactive bands. Experiments were repeated three times, and representative data are presented in the figures. Band intensities were quantified using ImageJ software (National Institutes of Health, Bethesda, MD).

### Kidney IHC.

For immunohistochemistry (IHC), kidneys were fixed in Mildform 10N (Wako Pure Chemical Industries, Saitama, Japan) for 8-OHdG and 4-hydroxy-2-nonenal (4-HNE) staining and in methacarn (60% methanol, 30% chloroform, and 10% acetic acid) for PGC-1α staining after embedding in paraffin. Sections of 3-μm thickness were incubated with the appropriate primary antibody and a Vectastain Elite ABC HRP kit (Vector Laboratories, Burlingame, CA) and developed by incubation with ImmPACT diaminobenzidine (DAB) peroxidase substrate (Vector Laboratories). Signal intensity was quantified using a Mantra quantitative pathology workstation with inForm image analysis software (PerkinElmer Japan, Yokohama, Japan). This software can recognize the cells with nuclear staining automatically and calculate the signal intensity of DAB staining per cell. Over 50 normal tubular and cyst epithelial cells were quantified with respect to signal intensity.

### Mitochondrial morphology and superoxide quantification.

To analyze mitochondrial morphology, WT 9-7 and RCTEC-LTA cells were stained with MitoTracker Green FM (M7514; Life Technologies, Grand Island, NY), and WT 9-12 and RCTEC-DBA cells were stained with MitoTracker Red FM (M22425; Life Technologies). Briefly, 1 mM stock solutions of MitoTracker solutions were diluted with DMEM to a 50 nM working concentration and incubated with cells for 15 min under growth conditions. A BZ-9000 fluorescence microscope (Keyence, Osaka, Japan) was used for observation. Automated quantification of mitochondrial morphology was done using ImageJ software. The mean area/perimeter ratio was employed as an index of mitochondrial interconnectivity, with inverse circularity used as a measurement of mitochondrial elongation and validated using well-characterized mediators of mitochondrial fission and fusion. Increased fluorescence of MitoSOX Red (M-36008; Molecular Probes, Eugene, OR) was used as an assay for mitochondrial superoxide production as previously described (60). Cells were incubated with 2.5 μM MitoSOX Red for 10 min in the dark at 37°C and then rinsed three times with phosphate-buffered saline prior to fluorescence measurement using a BZ-9000 fluorescence microscope (Keyence). The fluorescence intensity of each randomly selected cell was quantified using ImageJ software as previously described (61). Results are expressed as compiled means ± standard deviations from at least three independent experiments, representing relative fluorescence intensity normalized to controls.

### Cell analysis.

Oxidative stress, mitochondrial membrane potential, and cell counts were evaluated using a Muse oxidative stress kit, MitoPotential kit, and count and viability kit, respectively, on a Muse cell analyzer (Merck Millipore, Billerica, MA), and assays were performed according to the manufacturer's instructions. The Muse oxidative stress kit provides the relative percentages of ROS-negative and -positive cells, based on the intracellular detection of superoxide radicals and using an oxidative stress reagent based on dihydroethidium. The MitoPotential assay utilizes MitoPotential dye, a cationic, lipophilic dye that detects changes in mitochondrial membrane potential, with 7-aminoactinomycin D (7-ADD) used as an indicator of cell death.

### mtDNA sequence analysis.

mtDNA was isolated from cultured cells using a mitochondrion isolation kit (Pierce Biotechnology, Rockford, IL) and a NucleoSpin tissue kit (TaKaRa Bio). Primer pairs designed to generate overlapping mtDNA fragments are listed in Table 3. We used 10 ng of mtDNA for quantitative real-time PCR using Kapa SYBR fast universal 2× qPCR master mix (Kapa Biosystems) run on a CFX96 system (Bio-Rad). The PCR protocol consisted of 95°C for 30 s followed by 40 cycles at 95°C for 5 s, annealing at 59°C for 30 s, and a 72°C extension for 3 min, accompanied by real-time data collection. PCR products were sequenced by Eurofins Genomics (Tokyo, Japan), and mtDNA sequences were identified.

**TABLE 3:** Primers for analysis of mtDNA sequence

Mitochondrial DNA fragment (nucleotide positions)	Primer sequence, 5′→3′	
	Forward	Reverse
1 (47–1704)	GCATTTGGTATTTTCGTCTGGGG	AGTAAGGTGGAGTGGGTTTGG
2 (755–1914)	GCATCAAGCACGCAGCAAT	CTGGTTTCGGGGGTCTTAGC
3 (1616–3182)	ACACAAAGCACCCAACTTACAC	TATCATTTACGGGGGAAGGCG
4 (2414–3564)	CCTCACTGTCAACCCAACAC	TCATAGTAGAAGAGCGATGGTG
5 (3125–5491)	ACGAAAGGACAAGAGAAATAAGGC	GGGGAGATAGGTAGGAGTAGCG
6 (3878–5468)	CAGAGACCAACCGAACCCC	GGTAAGGGCGATGAGTGTGG
7 (4950–7140)	TCCATCATAGCAGGCAGTTGAG	GGATTTTGGCGTAGGTTTGGTC
8 (6048–7380)	GGTAACGACCACATCTACAACG	CCAGGTTTATGGAGGGTTCTTC
9 (6416–7288)	AAAACCCCCTGCCATAACCC	AGAGAAATGAATGAGCCTACAGATG
10 (6959–8947)	CCTGACTGGCATTGTATTAGCA	GTATGGGGATAAGGGGTGTAGG
11 (7772–8939)	ACCGTCTGAACTATCCTGCC	ATAAGGGGTGTAGGTGTGCC
12 (8538–10147)	TCTGTTCGCTTCATTCATTGCC	TAGCCGTTGAGTTGTGGTAGTC
13 (9273–9792)	TCAGCCCTCCTAATGACCTCC	TTGAGCCGTAGATGCCGTC
14 (9499–11209)	TCTGAGCCTTTTACCACTCCAG	TAGGAAGTATGTGCCTGCGTTC
15 (10291–11166)	CCCTACAAACAACTAACCTGCC	ATCGGGTGATGATAGCCAAGG
16 (10830–12623)	GAATCAACACAACCACCCACAG	ACGAACAATGCTACAGGGATGA
17 (11597–12407)	ACAGACCTAAAATCGCTCATTGC	ACGAGGGTGGTAAGGATGGG
18 (11989–13568)	ATTTACCACAACACAATGGGGC	ATAGATAGGGCTCAGGCGTTTG
19 (12801–13778)	CAGTTGATGATACGCCCGAG	TTGTTTGGAAGGGGGATGCG
20 (13436–14765)	CTCTCACTTCAACCTCCCTCAC	TTTTGCGTATTGGGGTCATTGG
21 (13926–15454)	TAGCATCACACACCGCACAATC	AAGGAAGAGAAGTAAGCCGAGG
22 (14684–15633)	CGGACTACAACCACGACCAA	AGTAATAGGGCAAGGACGCC
23 (15264–16509)	CCACCCTCACACGATTCTTTAC	AGGAACCAGATGTCGGATACAG
24 (15977–16521)	CCACCATTAGCACCCAAAGC	TGACCCTGAAGTAGGAACCAGA
25 (16245–382)	CAACTCCAAAGCCACCCCTC	GGCTGGTGTTAGGGTTCTTTG

### siRNA-mediated depletion of PKD1 expression.

We used Hs_PKD1_2 (SI00006454, termed si*PKD1*-1) and Hs_PKD1_6 (SI03115560, termed si*PKD1*-2) as human *PKD1*-specific siRNAs. We used AllStar Neg siRNA AF 488 as the negative control of siRNA. These siRNAs were obtained from Qiagen (Venlo, The Netherlands). Cells were transfected with siRNAs by using Lipofectamine RNAiMAX transfection reagent obtained from Invitrogen (Carlsbad, CA) and analyzed at 48 h after transfection. Knockdown efficiency was confirmed by quantitative real-time PCR by using specific primers as listed in Table 4.

**TABLE 4:** Primers for analysis of human *PKD1* knockdown efficiency

Target	Primer sequence, 5′→3′	
	Forward	Reverse
si*PKD1*-1	CTCAATGCCTCCAACGCAGT	CAGCCAGCAGGATCTGAAAATG
si*PKD1*-2	GCTTCACTAGCTTCGACCAGG	TCGGCATAATGTCTTGCCAAAG
GAPDH	CCTCAACGACCACTTTGTCA	TTACTCCTTGGAGGCCATGT

### Intracellular Ca^2+^ concentration.

Cells were incubated with 2.5 μM Fura-2 AM (F015; Dojindo, Kumamoto, Japan) diluted with recording medium (20 mmol/liter HEPES, 115 mmol NaCl, 5.4 mmol/liter KCl, 0.8 mmol/liter MgCl_2_, 1.8 mmol/liter CaCl_2_, and 13.8 mmol/liter glucose [pH 7.4]) with 0.05% pluronic F-127 (P2443; Sigma-Aldrich) for 1 h at 37°C in 95% air–5% CO_2_. Fura 2-AM was excited at 340 nm and 380 nm, and emission at 510 nm was recorded using a 2300 EnSpire (PerkinElmer Japan). Data represent the results of three independent experiments performed in duplicate.

### Intracellular cAMP assay.

The cAMP concentration was measured using the enzyme immunoassay Parameter kit (KGE002B; R&D Systems, Minneapolis, MN) according to the manufacturer's instructions. Extracellular cAMP was not measured, and no correction was applied for cAMP released from cells. Absorbance values were measured using a 2300 EnSpire (PerkinElmer Japan) and converted to cAMP concentrations according to the manufacturer's instructions. Data represent the results of two independent experiments performed in duplicate, and all values were normalized to the values from controls.

### Calcineurin assay.

Cell lysates were prepared using reagents provided in the calcineurin cellular activity assay kit (BML-AK816; Enzo Life Science, Farmingdale, NY), and the assay was performed according to the manufacturer's instructions. Absorbance values were measured using a 2300 EnSpire and converted into the amount of PO_4_ released by calcineurin according to the manufacturer's instructions. All values were normalized to control values.

### NOS activity assay.

Intracellular NOS activity was measured using a nitrate/nitrite colorimetric assay kit (760871; Cayman Chemical, Ann Arbor, MI) according to the manufacturer's instructions. Absorbance values were measured using a 2300 EnSpire and converted into the amounts of nitrate and nitrite according to the manufacturer's instructions.

### BrdU flow cytometry.

Cells were analyzed by staining with bromodeoxyuridine (BrdU) and 7-ADD (BrdU flow kit; BD Pharmingen, Franklin Lakes, NJ) according to the manufacturer's instructions. The experiment was performed in triplicate, and samples were analyzed by flow cytometry (BD FACSVerse; BD Pharmingen).

### OCR.

An XF96 extracellular flux analyzer (Agilent Technologies, Santa Clara, CA) was used to determine intracellular bioenergetic profiles. The cell number seeded onto each well of an XF96 cell culture microplate was 1.0 × 10^4^ cells/well. The oxygen consumption ratio (OCR) was assessed in glucose-containing XF base medium (25 mM glucose, 2 mM pyruvate glutamine, and 1 mM pyruvate) according to the manufacturer's instructions. The concentrations of oligomycin, carbonyl cyanide-4-(trifluoromethoxy)phenylhydrazone (FCCP), and antimycin A/rotenone were 1, 0.5, and 1 μM, respectively. XF24 was also used to determine the OCR in cells treated with H-89. The cell number seeded onto each well of an XF24 cell culture microplate was 2.5 × 10^4^ cells/well.

### Chemical reagents.

MitoQuinone {phosphonium [10-(4,5-dimethoxy-2-methyl-3,6-dioxo-1,4-cyclohexadien-1-yl)decyl] triphenyl-mesylate} (MitoQ) was first designed as an antioxidant intended to block mitochondrial oxidative damage by preventing lipid peroxidation (62). MitoQ was kindly provided by M. Murphy (MRC Mitochondrial Biology Unit, Cambridge, UK).

### Statistical analysis.

All data are presented as means ± standard deviations. Data for two groups were analyzed by the unpaired *t* test, and those for more than two groups were compared by analysis of variance with a Tukey-Kramer test. A *P* value of <0.05 was considered statistically significant. JMP Pro software version 12.2.0 (SAS Institute Japan, Tokyo, Japan) was used for data analysis.
